# Multimodal Imaging Evaluation of Myocardial Involvement in Rare Oculoleptomeningeal Hereditary Transthyretin Amyloidosis

**DOI:** 10.1016/j.jaccas.2025.104034

**Published:** 2025-07-16

**Authors:** Moritz J. Hundertmark, Martin Zinkernagel, Tatiana Brémova-Ertl, Pascal Escher, Anina Bauer, Federico Caobelli, Yara Banz, Andreas Flammer, Michele V. Martinelli, Christoph Gräni

**Affiliations:** a Department of Cardiology, Inselspital, University Hospital of Bern, University of Bern, Bern, Switzerland; b Department of Ophthalmology, Inselspital, University Hospital of Bern, University of Bern, Bern, Switzerland; c Department of Neurology, Inselspital, University Hospital of Bern, University of Bern, Bern, Switzerland; d Department of Human Genetics, Inselspital, University Hospital of Bern, University of Bern, Bern, Switzerland; e Department of Nuclear Medicine, University Hospital of Bern, University of Bern, Bern, Switzerland; f Institute of Tissue Medicine and Pathology, University of Bern, Bern, Switzerland; g Department of Cardiology, University Hospital Zurich, University of Zurich, Zurich, Switzerland

**Keywords:** amyloidosis, CM-ATTR, p.Ile127Met, rare disease, vATTR

## Abstract

**Background:**

In hereditary (variant) transthyretin amyloidosis (vATTR), cardiomyopathy leads to worse outcomes. Of 140 identified TTR gene mutations, few are associated with oculoleptomeningeal amyloidosis. It remains unclear whether these mutations are linked to a cardiac phenotype.

**Case Summary:**

A 59-year-old man from Bosnia and Herzegovina initially presented with transient vision loss and was ultimately diagnosed with rare p.Ile127Met vATTR. We describe a detailed investigation of myocardial involvement harnessing multimodal cardiovascular imaging.

**Discussion:**

No previous reports have specifically addressed the cardiac phenotype of oculoleptomeningeal amyloidosis. Our paper depicts a mild cardiac phenotype for the p.Ile127Met variant highlighting the need for awareness among cardiologists and emphasizing the necessity for close patient monitoring and expertise in evolving disease-modifying treatments.

**Take-Home Messages:**

Early diagnosis and risk stratification in patients with vATTR are challenging. Cardiologists need an increased awareness because they may encounter patients with seemingly rare vATTR, such as the p.Ile127Met variant discussed here.

**Visual Summary:**
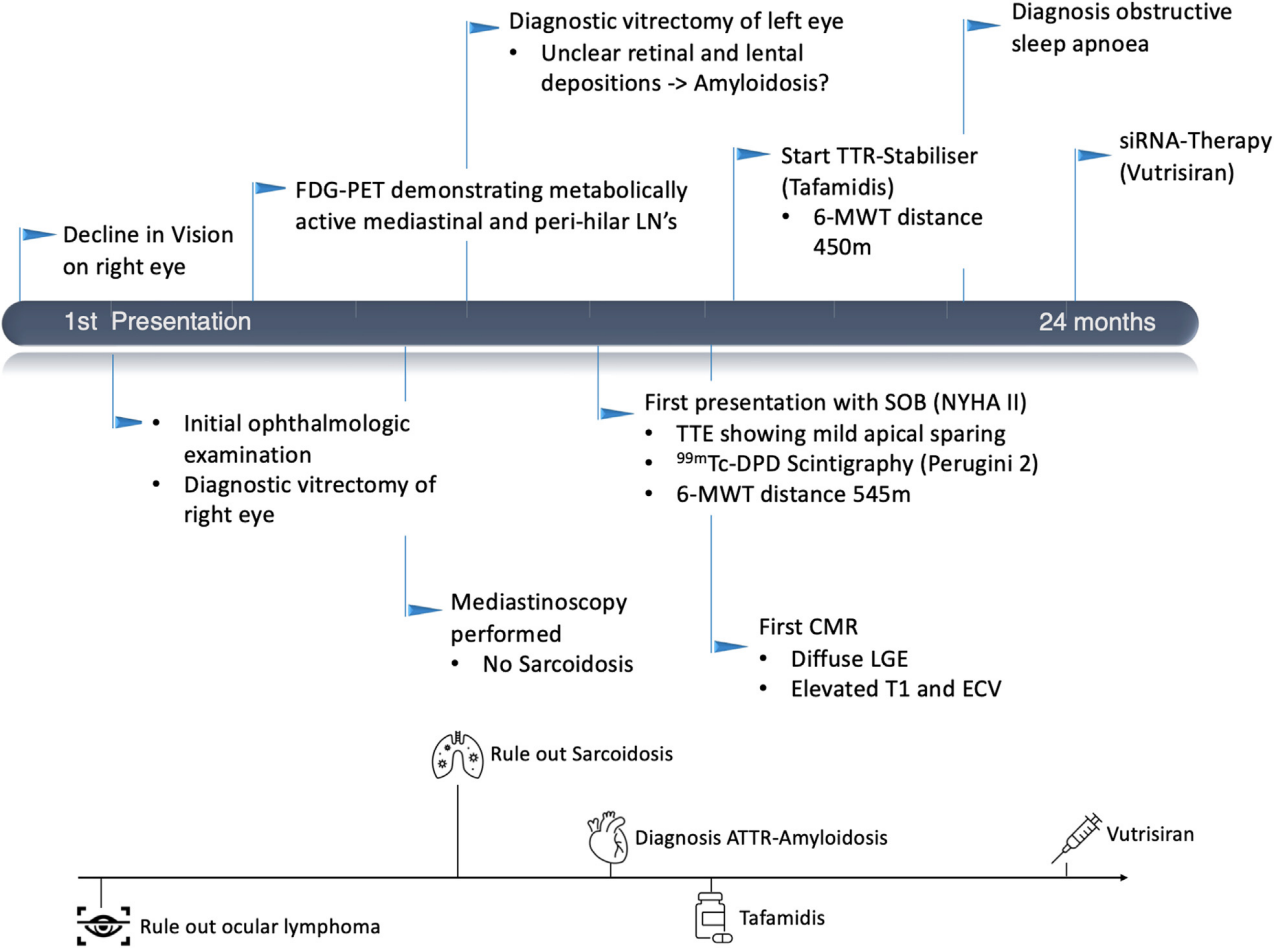
Case Summary Simplified overview of the diagnostic tests and clinical events leading to diagnosis of hereditary transthyretin amyloidosis (vATTR) as well as commencing disease-modifying therapy of a transthyretin (TTR) stabilizer (tafamidis) and small interfering RNA (siRNA) (vutrisiran) (upper pane). Lower pane: selected key milestones in making the diagnosis of vATTR and disease-specific treatment for vATTR cardiomyopathy. 6-MWT = 6-minute walking test; ^99m^Tc-DPD, technetium-99m–labeled 3,3-diphosphono-1,2-propanodicarboxylic acid; CMR = cardiovascular magnetic resonance; ECV = extracellular volume; FDG-PET = fluorodeoxyglucose positron-emitting tomography; LGE = late gadolinium enhancement; LN = lymph node; SOB = shortness of breath; TTE = transthoracic echocardiogram.

**Take-Home Messages**

• Early diagnosis and effective risk stratification in patients with vATTR remain challenging for cardiologists.
• A multidisciplinary approach is essential to identify at-risk patients
• Increased awareness of potential cardiac involvement in seemingly extracardiac vATTR mutations, such as the oculoleptomeningeal p.Ile127Met variant discussed here, is crucial for cardiologists.

## History of Presentation

A 59-year-old man from Bosnia and Herzegovina presented to our University Hospital's ophthalmology department for evaluation of intermittent decline in vision, predominantly on the right eye, over a period of 2 months. Before this, there had been episodes of unexplained fatigue and vertigo. Cursory physical examination was unremarkable, whereas the ophthalmologic examination revealed a bilateral uveitis with a vitreous haze, predominantly on the right eye. Notably, the patient's wife stated that her husband's sister had presented in a Bosnian hospital with similar issues previously, with other distant family members (cousins) reportedly having had “eye problems” when they were quadragenarians or pentagenarians.

## Past Medical History

There were no relevant cardiovascular events in the patient's past medical history although he used to smoke before quitting last year (estimated 60 pack-years). He previously reported intermittent vertigo for which oral betahistine (16 mg once daily) was prescribed as required. Other than this, benign prostate hyperplasia was reported for which oral tamsulosin (0.4 mg once daily) was prescribed.

## Differential Diagnosis

Because of the amount of vitreous haze observed during the eye examination and resulting difficulty in performing a full fundoscopy, a diagnostic vitrectomy of the right eye was scheduled after the initial examination. It was considered that uveitis may masquerade a more serious underlying condition.^1^ In fact, after vitrectomy, protein electrophoresis of the vitreous humor revealed a small increase in the gamma globulin light chains, leading to a suspicion of intraocular lymphoma. Subsequently, the patient was referred to the local multidisciplinary lymphoma board for further testing and discussion.

## Investigations

To investigate a possible intraocular lymphoma, cerebral magnetic resonance imaging, detailed blood analysis and lumbar puncture were performed, which eventually could not confirm the suspicion. Because uveitis can be a sign of sarcoidosis, a fluorodeoxyglucose positron-emitting tomography was recommended. As this demonstrated metabolically active mediastinal and perihilar lymph nodes, biopsies were scheduled; however, anatomical considerations rendered bronchoscopic fine needle biopsies difficult. Consequently, a mediastinoscopy was performed with histopathologic analyses of excised lymph nodes thereafter. These did not show any signs of sarcoidosis and were not pathognomonic for any other disease.

**Figure 1:**
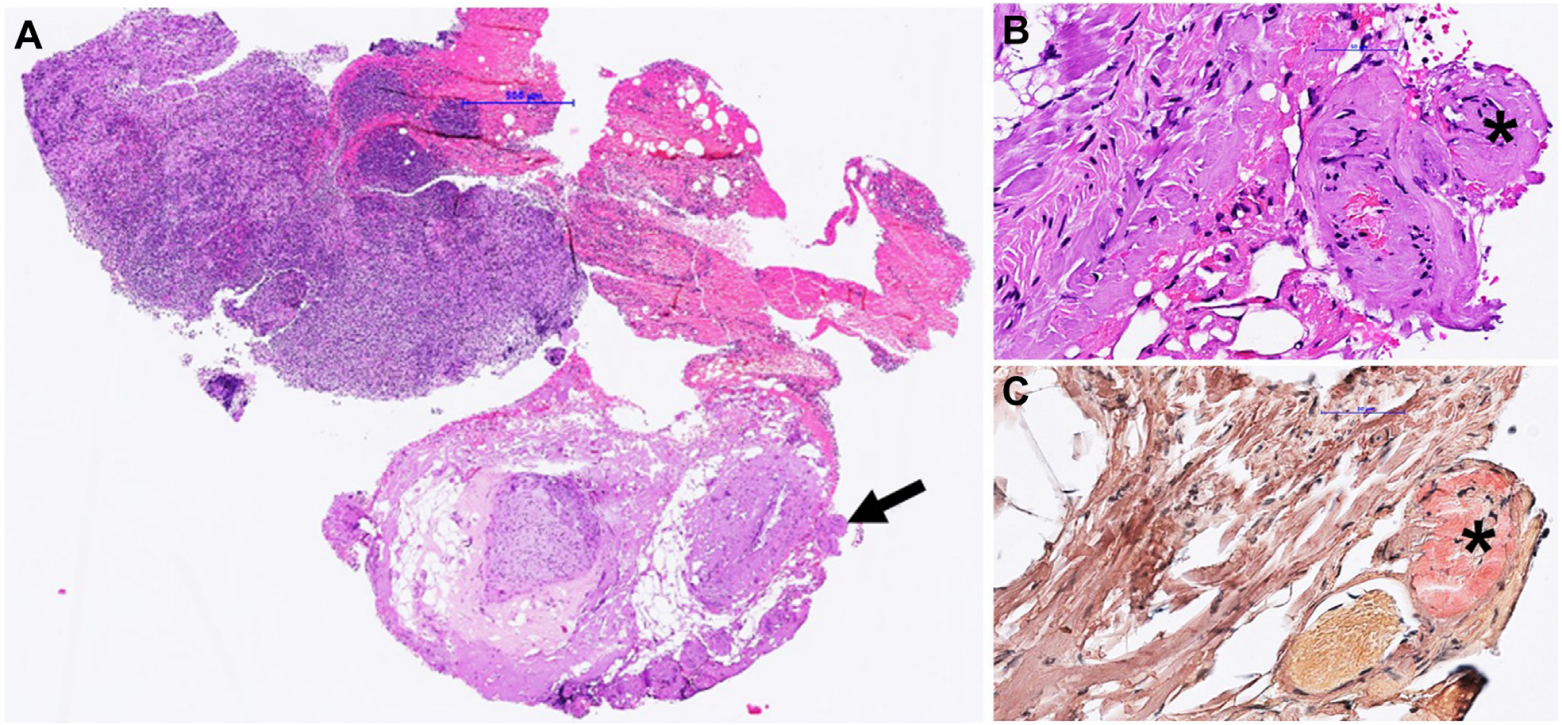
Mediastinal Lymph Node Tissue and Adjacent Vascularized Soft Tissue Overview (A) and details (B, C) of mediastinal lymph node tissue and adjacent vascularized soft tissue. A hematoxylin and eosin stain (A, B) shows lymphoid tissue (upper left) and adjacent soft tissue. The black arrow marks an area of hyalinized tissue in a perivascular space. (C) (Congo stain) A Congo red area (asterisk), corresponding to the hyalinized area (asterisk in ‘B’) with green birefringence (not shown), compatible with amyloid deposits. Typing using specific antibodies in a reference laboratory revealed ATTR amyloid deposits (data not shown).

**Figure 2:**
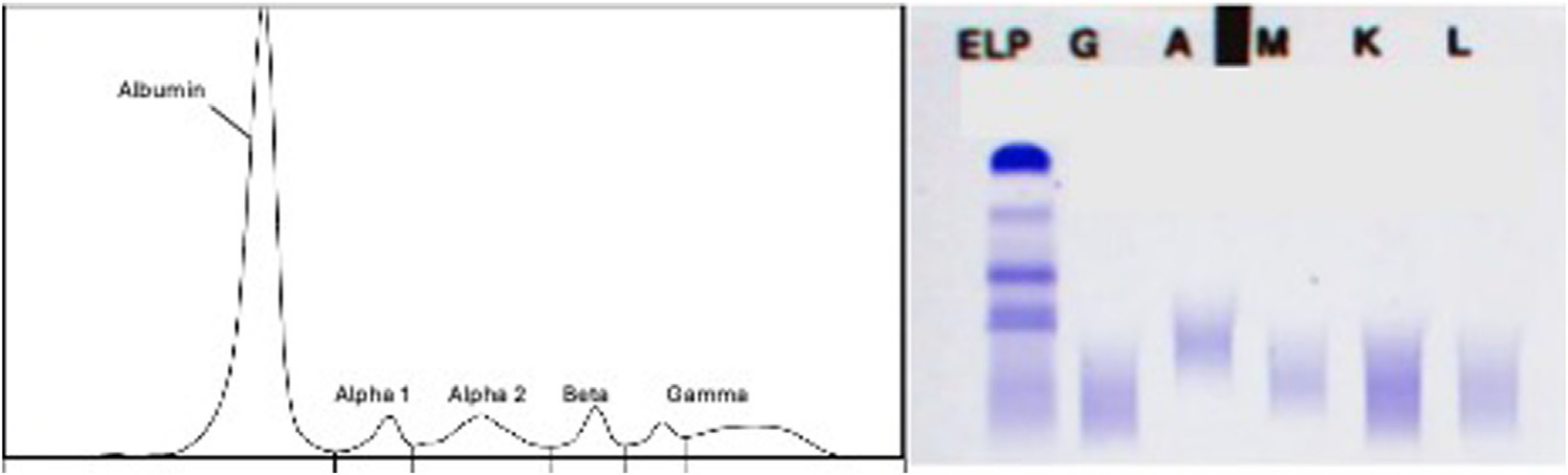
Serum M Protein Tests Results serum M protein tests of the patient. Left panel: serum protein electrophoresis showing a mild unspecific hypogammaglobulinemia with otherwise normal electrophoresis pattern of albumin, alpha 1–protein, alpha 2–protein, and beta-protein fractions. Right panel: immune fixation electrophoresis (ELP) showing no signs of monoclonal light chains. A = immunoglobulin alpha; G = immunoglobulin gamma; K = immunoglobulin kappa; L = immunoglobulin lambda; M = immunoglobulin M.

**Figure 3:**
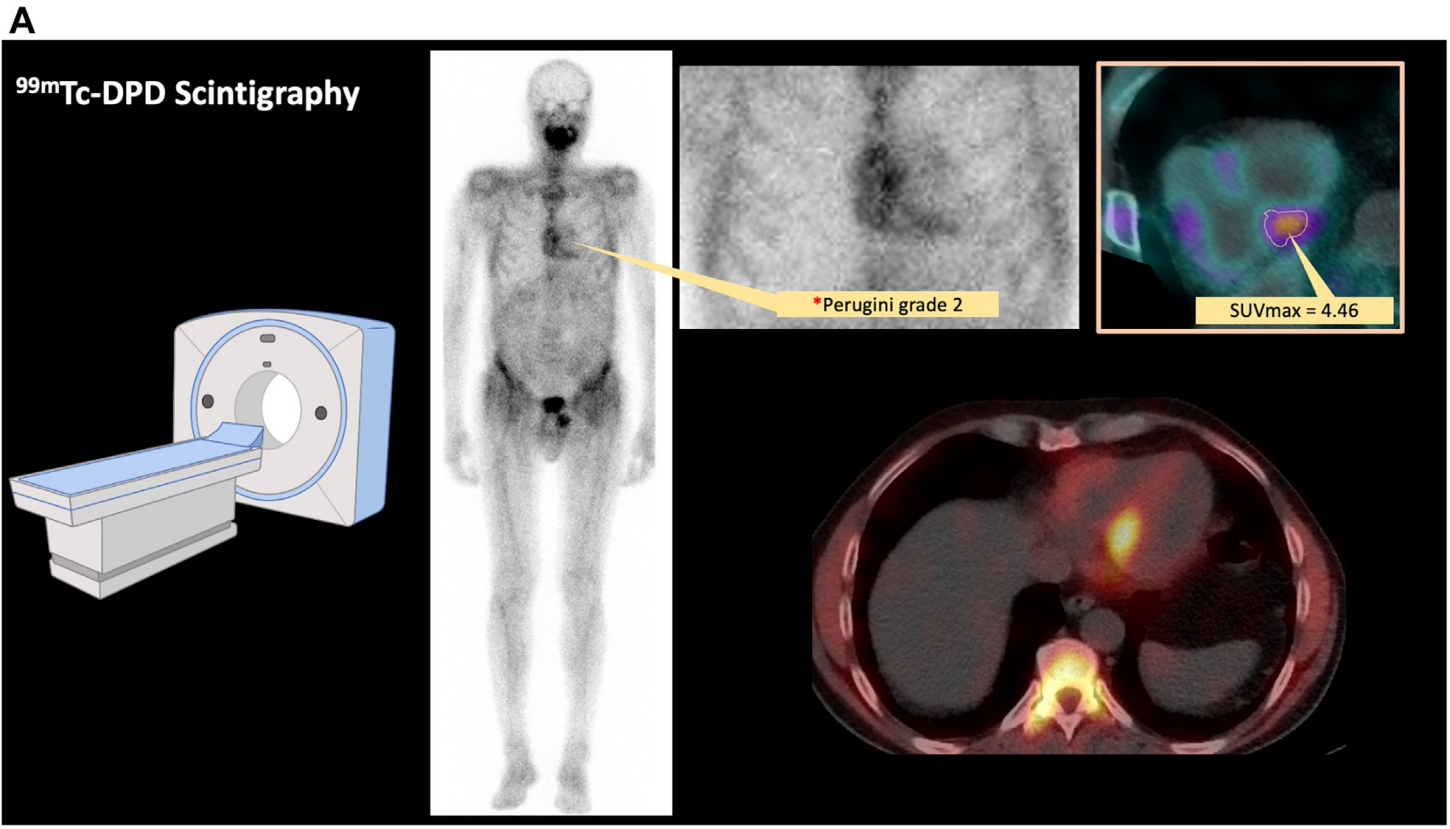

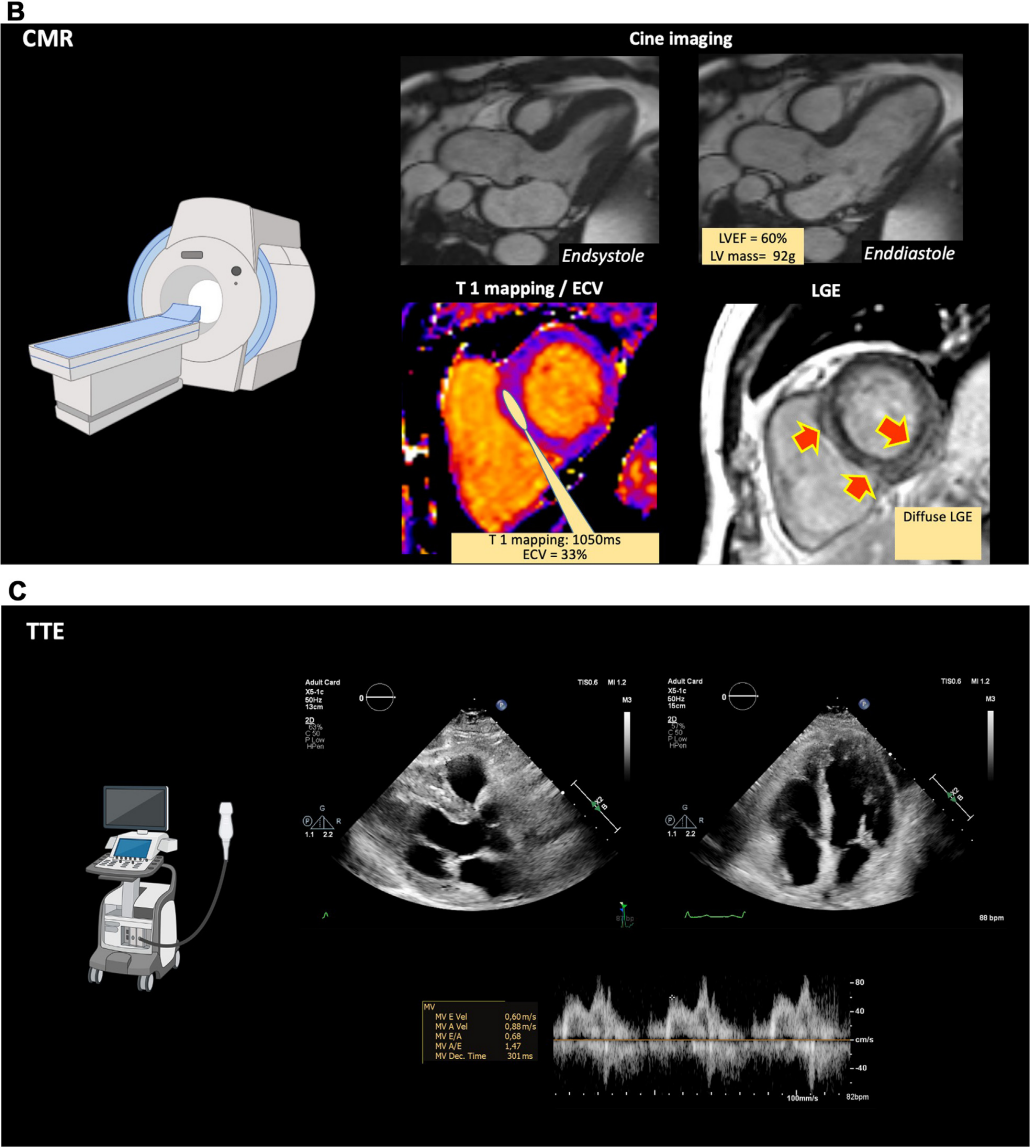
Multimodality Cardiac Imaging Findings Results from multimodality cardiac imaging investigations. (A) Scintigraphy using technetium-99m–labeled 3,3-diphosphono-1,2-propanodicarboxylic acid (^99m^Tc-DPD) showing myocardial tracer uptake consistent with a grade 2 as described previously by Perugini.^2^ The standardized maximal uptake value at 1 hour (SUVmax) depicted in the upper right of the figure provides a quantitative assessment of cardiac amyloid burden and has previously been described to correlate well with measures of tissue characterization on cardiovascular magnetic resonance (CMR).^3^ Axial single-photon emission computed tomography/computed tomography image showing tracer uptake in the myocardial septum. (B) Upper row depicting 3-chamber views obtained from CMR imaging in end-diastole and end-systole with normal left ventricular ejection fraction (LVEF) and left ventricular mass (LV mass). Bottom panel presenting the findings from tissue characterization methods. Assessment of parametric mapping (T1) and calculation of extracellular volume (ECV) revealed elevated T1-values and mildly elevated ECV. Late gadolinium enhancement (LGE) showing sub-endocardial and epicardial diffuse enhancement consistent with amyloid fibril deposition. (C) Representative images of parasternal long axis (upper-left pane) and 4-chamber (upper-right pane) views on transthoracic echocardiography. Pulsed wave Doppler measurements of inflow velocities across the mitral valve showing abnormal diastolic relaxation with a ratio of early and late diastolic filling across the mitral valve of 0.68 and a prolonged mitral E-wave deceleration time of 301 milliseconds.

**Figure 4:**
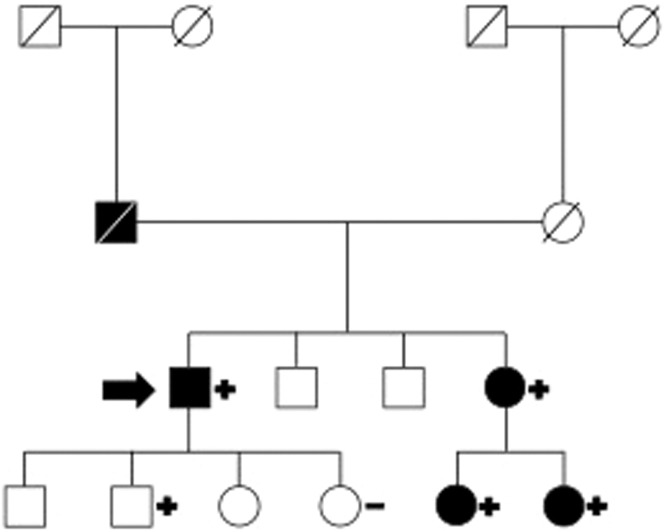
Patient Family Pedigree Pedigree of the family affected by autosomal dominant hereditary transthyretin amyloidosis caused by a heterozygous pathogenic c.381T>G p.Ile127Met variant in transthyretin. The patient discussed in the main text is marked by an arrow. Symbols of individuals in black indicate clinical presentation in keeping with typical symptoms for the p.Ile127Met variant. The “plus” symbols represent individuals carrying the genetic variant, whereas the “minus” symbol indicates a negative genetic test result. Deceased individuals are marked by a line in the respective rectangle or circle.

Because of the scarcity of the material from the initial vitrectomy of the right eye, the decision was made to perform an additional diagnostic vitrectomy of the left eye. In this examination, approximately 1 year after first presentation, retinal and lenticular deposits were observed, which raised suspicion for amyloidosis. The previously attained bioptic material entailed a small amount of subcutaneous fat, and a Congo red stain revealed pathognomonic deposits suggestive of transthyretin amyloidosis (ATTR) (Figure 1). Exclusion of light-chain amyloidosis was confirmed via normal serum protein and immune fixation electrophoresis (Figure 2). After a formal cardiology referral, a transthoracic echocardiogram (TTE) demonstrated preserved systolic function but mild diastolic dysfunction (Figure 3C). A full body bone scintigraphy with technetium-99m–labeled 3,3-diphosphono-1,2-propanodicarboxylic acid discovered myocardial tracer uptake (Perugini grade 2) (Figure 3A) with an elevated standardized myocardial tracer uptake of 4.46, highly suggestive for ATTR cardiomyopathy. Despite normal biventricular dimensions, overall mass and systolic function (left ventricular ejection fraction 60%, right ventricular ejection fraction 53%), cardiovascular magnetic resonance confirmed myocardial involvement with an elevated T1 time and an increased extracellular volume fraction of 33%. In addition, tissue characterization using late gadolinium enhancement demonstrated diffuse enhancement (Figure 3B). Lastly, genetic testing from peripheral blood revealed a missense mutation in the transthyretin (TTR) gene, leading to a replacement of thymine with guanine at nucleotide 381 (c.381T>G), ultimately substituting isoleucine for methionine in the TTR protein in amino acid 127 (p.Ile127Met), leading to the final diagnosis of a variant transthyretin amyloidosis (vATTR). An extended family history elucidated that the patient's father died of unknown “heart problems” at age 56 years, whereas his mother died of natural causes at age 86 years. The patient has 2 brothers and a sister, of whom only the latter underwent genetic testing after the decline in vision and vertigo as initial symptoms in her fifth decade and has recently been diagnosed with vATTR of the same genotype. His sister has 2 daughters who have been tested and diagnosed with the p.Ile127Met variant after a recent history of fatigue and vertigo. The patient's children (2 daughters and 2 sons) have not reported any suggestive symptoms thus far. One of the patient's daughters has decided to undergo genetic testing but was not diagnosed as a mutation carrier, whereas the patient's son revealed a positive genotype. We have summarized the patient's family pedigree in Figure 4.

## Management

The patient was referred to the cardiology-led amyloidosis clinic, and the initial diagnostic work-up included a 6-minute walking test (6-MWT), which showed a walking distance of 540 m, while highly sensitive troponin and N-terminal pro–B-type natriuretic peptide were within the normal range. A few months later, despite a normal N-terminal pro–B-type natriuretic peptide level (80 pg/mL), the patient presented with worsening fatigue and NYHA functional class II shortness of breath. A repeated 6-MWT demonstrated a decline in the walking distance by 90 m (to now 450 m) while the patient's vertigo had worsened. A repeated TTE did not corroborate any changes compared with the previous echocardiogram. After discussion of the case in our multidisciplinary amyloidosis board, we began disease-modifying treatment with tafamidis, an established TTR stabilizer, which had previously shown efficacy in patients with vATTR for cardiopathy and neuropathy.^4^

## Outcome and Follow-Up

**Figure 5:**
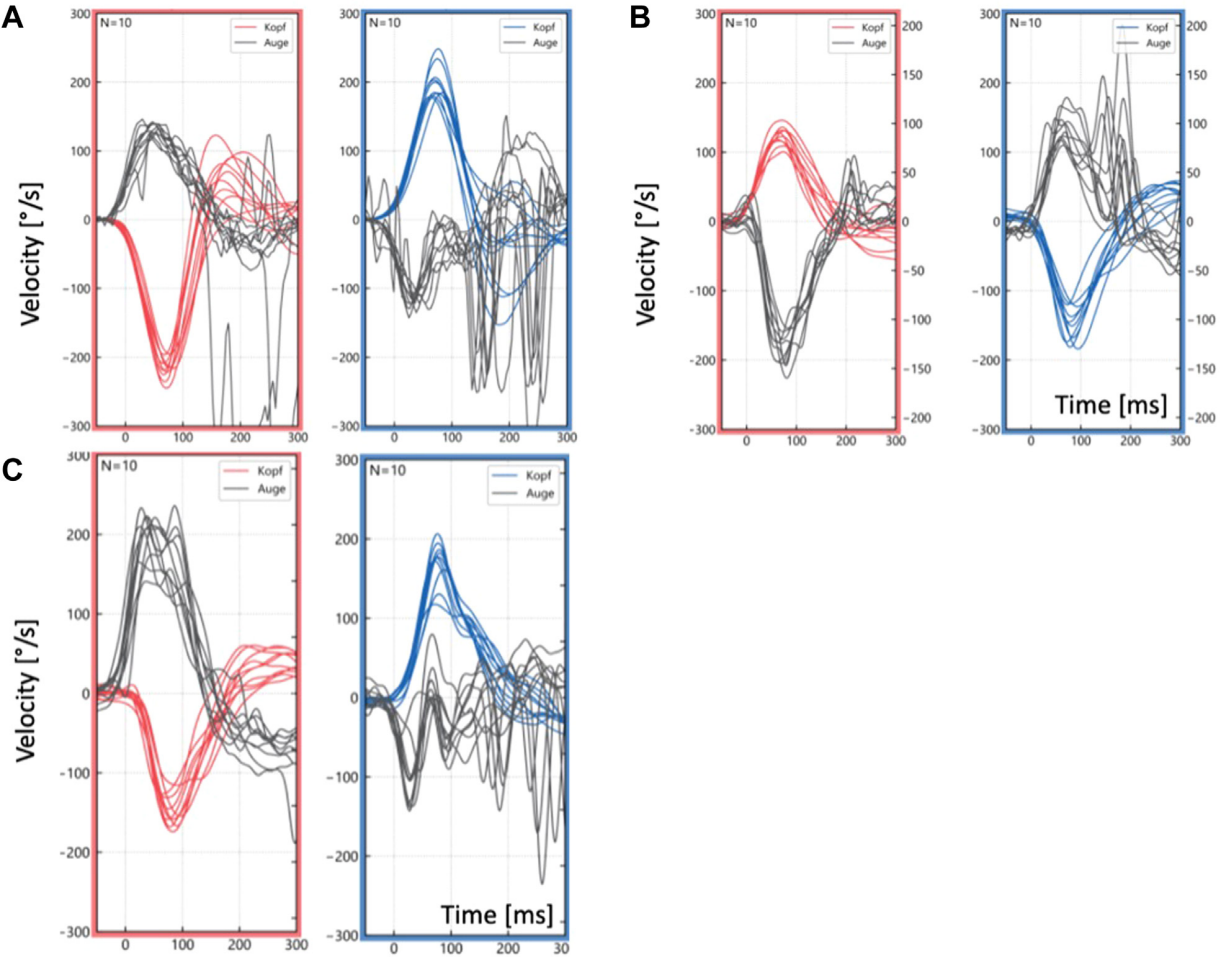
Video Head Impulse Test Examination (A) Head-impulse test of the right (red) and left (blue) semicircular canal. The right horizontal canal gain (eye velocity vs head velocity) yielded 0.58, and the left horizontal canal gain yielded 0.32. Note the catch-up saccades on the left side. (B) Head-impulse test of the right anterior (red) and left posterior (blue) semicircular canal with the gain of right anterior canal 1.29 and left horizontal canal 0.96, with profound corrective saccades on the left side. (C) Head-impulse test of the right posterior (red) and left anterior (blue) semicircular canal with the gain of right posterior canal 1.37 and left horizontal canal 0.22, again with corrective, overt saccades left. The red and blue lines indicate the head movement and the black lines the eye movement.

A subsequent examination revealed worsening autonomic dysfunction with increased vertigo, reduced walking distance in the 6-MWT, and progressive fatigue. Thus, the patient was deemed a poor responder to tafamidis and subsequently administered subcutaneous vutrisiran, a novel RNA interference molecule, inducing a rapid reduction of serum TTR levels.^5^ On follow-up, the patient was diagnosed with bilateral vestibulopathy of all semicircular canals (left > right side) explaining his dizziness and oscillopsia (Figure 5). In addition, a carpal tunnel syndrome was noted on the left side.

## Discussion

In the current case, we present a rare p.Ile127Met missense mutation leading to oculoleptomeningeal amyloidosis (OLMA), which has only been described in 3 individuals previously, 2 of those affected being of Bosnian descent.^6^, ^7^, ^8^ Former reports lacked a thorough cardiac assessment in affected patients. To the best of our knowledge, this is the first comprehensive evaluation of the cardiac phenotype in individuals with the p.Ile127Met variant, supported by advanced multimodality cardiac imaging, including scintigraphy, TTE, and cardiovascular magnetic resonance.

TTR is a tetrameric protein consisting of 127 amino acids, whose main function is to bind and distribute retinol and thyroxine. The majority of TTR (90%) is synthesized in the liver, but a certain fraction (10%) is produced in the choroid plexus and retina.^9^ More than 140 vATTR mutations have been described; however, a systematic classification of associated phenotypes and detailed assessments of involved organ systems are lacking.^10^ As described earlier, the p.Ile127Met variant leads to an early-onset OLMA, with central nervous system dysfunction, neuropathy, and vision disturbances; yet, it remains elusive to what extent the heart is affected. Our case presents evidence of a mild cardiac phenotype, corroborated by multimodality imaging. As such, affected patients need cardiologic surveillance, and cardiologists need proficiency for managing patients with OLMA. Notably, regulation of TTR production may differ in the respective locations, and it remains elusive why amyloidogenic TTR fibrils accumulate in certain organs.^11^ Tafamidis is a kinetic stabilizer of the tetrameric TTR protein and prevents the dissociation into monomers that eventually misfold and aggregate in amyloidogenic fibrils, for example, in the heart.^12^ Individual mutant TTR proteins each differ in their dissociation kinetics, and regulation of their expression may differ; hence, certain TTR variants may possibly be less susceptible to stabilizers. Nevertheless, because of exclusion of patients with OLMA in HELIOS-B^5^ and virtually all trials of available ATTR disease-modifying treatments, it is unknown whether novel treatments (so-called silencers such as vutrisiran or patisiran) will demonstrate a similar benefit in patients with OLMA compared with those with wild-type ATTR and more common vATTR (such as p.Val50Met and p.Val142Ile) and whether a combination therapy provides improved efficacy over monotherapy. The patient presented here has only recently received the first silencer dose (vutrisiran); hence, it is currently not feasible to assess its efficacy, but caution is warranted because vutrisiran is unable to cross the blood-brain barrier and a different patient in a previous report of the p.Ile127Met variant experienced worsening symptoms while undergoing treatment with patisiran.^8^ Because the cardiac disease progression of the p.Ile127Met variant remains elusive, it is advisable to assess treatment response and cardiac function regularly. Although the optimal surveillance strategy for asymptomatic mutation carriers remains to be determined, our approach is to perform baseline echocardiography and neurology assessments and perform annual outpatient follow-ups in our multidisciplinary amyloidosis center. Further investigations such as advanced cardiac imaging and rhythm monitoring depend on symptom presentation.

The observed vestibular dysfunction may well be associated with vATTR; however, whether vATTR is causally responsible is currently unknown. Vestibular dysfunction has previously been described in Alzheimer disease, where beta-amyloid deposition in the central nervous system is the disease mechanism. There has been growing appreciation that vascular deposition of TTR amyloid fibrils may lead to angiopathy and thus vestibular dysfunction. Although this remains to be investigated and is currently hypothetical, cases like ours may fuel this debate and accordingly provoke new research efforts into this special population.

## Conclusions

Here, we report the first detailed assessment of a cardiac phenotype in rare OLMA caused by a p.Ile127Met missense mutation. Because unspecific symptoms such as vertigo frequently incur cardiology assessment, increasing awareness for rare vATTR phenotypes, such as the p.Ile127Met mutation, is required in patients from certain ethnic backgrounds—in our case Bosnian—with suspicious family histories. Response to established therapies for the p.Ile127Met variant may differ from more common vATTR mutations (eg, p.Val50Met, p.Val142Ile) as current therapies target hepatic TTR production and do not cross the blood-brain barrier at all (vutrisiran and patisiran) or in relevant quantities (tafamidis). Hence, patients should be closely monitored for signs of disease progression.

## Funding Support and Author Disclosures

The University Hospital Bern receives funding for the SWISS-CARE Amyloidosis registry from 10.13039/100004319Pfizer and AstraZeneca. The authors have reported that they have no relationships relevant to the contents of this paper to disclose.
